# OsOLP1 contributes to drought tolerance in rice by regulating ABA biosynthesis and lignin accumulation

**DOI:** 10.3389/fpls.2023.1163939

**Published:** 2023-05-30

**Authors:** Jianpei Yan, Vincent Ninkuu, Zhenchao Fu, Tengfeng Yang, Jie Ren, Guangyue Li, Xiufen Yang, Hongmei Zeng

**Affiliations:** State Key Laboratory for Biology of Plant Diseases and Insect Pests, Institute of Plant Protection, Chinese Academy of Agricultural Sciences, Beijing, China

**Keywords:** OsOLP1, drought tolerance, rice, abscisic acid, lignin

## Abstract

Rice, as a major staple crop, employs multiple strategies to enhance drought tolerance and subsequently increase yield. Osmotin-like proteins have been shown to promote plant resistance to biotic and abiotic stress. However, the drought resistance mechanism of osmotin-like proteins in rice remains unclear. This study identified a novel osmotin-like protein, OsOLP1, that conforms to the structure and characteristics of the osmotin family and is induced by drought and NaCl stress. CRISPR/Cas9-mediated gene editing and overexpression lines were used to investigate the impact of OsOLP1 on drought tolerance in rice. Compared to wild-type plants, transgenic rice plants overexpressing OsOLP1 showed high drought tolerance with leaf water content of up to 65%, and a survival rate of 53.1% by regulating 96% stomatal closure and more than 2.5-fold proline content promotion through the accumulation of 1.5-fold endogenous ABA, and enhancing about 50% lignin synthesis. However, OsOLP1 knockout lines showed severely reduced ABA content, decreased lignin deposition, and weakened drought tolerance. In conclusion, the finding confirmed that OsOLP1 drought-stress modulation relies on ABA accumulation, stomatal regulation, proline, and lignin accumulation. These results provide new insights into our perspective on rice drought tolerance.

## Introduction

Drought is a significant environmental stress that adversely affects plant growth, development, and rhythm. Climate uncertainty leads to water deficits, and crops coordinate diverse signaling cascades and osmotic adjustments to resist drought, maintain extrinsic metabolism, and increase production to ensure food supply worldwide (Hu and Xiong, 2014). Rice is an essential commodity that is consumed by about half of the global population (Wei and Huang, 2019). However, rice is also an extremely water-consuming crop owing to its tiny root architecture and rapid stomatal aperture. Drought constrains rice growth and yield enhancement (Sahebi et al., 2018).

When plants suffer from water shortages in the external environment, signaling commences adopting various strategies to avoid physiological water imbalances in the osmotic potential of cells, thereby preventing drought damage (Hu and Xiong, 2014; Gupta et al., 2020). Abscisic acid (ABA) is an essential regulatory hormone that is responsive to adverse abiotic factors. Under drought conditions, ABA signals activate downstream resistance components that participate in water regulation (Gupta et al., 2020). Plants mainly lose water through transpiration *via* stomata on their leaves. Stomata contain two specialized guard cells whose density and movement determine the rate of transpiration. Previous studies have demonstrated that plants can enhance drought tolerance by mediating water utilization through stomata, as evidenced by the overexpression of *ERECTA*, *ENHANCED DROUGHT TOLERANCE1/HOMEODOMAIN GLABROUS11* (*EDT1/HDG11*) in Arabidopsis, and *stress-induced protein kinase gene 1* (*SIK1*), and *transcription factor 1-like* (*TF1L*) from rice (Masle et al., 2005; Ouyang et al., 2010; Zhu et al., 2016; Bang et al., 2019). The accumulation of intracellular solutes decreases osmotic potential, allowing plants to maintain turgor stress. Proline can act as an osmolyte to cope with unfavorable conditions faced by plants. Plant lignification can reflect the degree of drought tolerance, and synthetic lignin content can express the degree of lignification (Bang et al., 2019; Bang et al., 2022). Lignin helps maintain the osmotic balance of cells by controlling osmosis and transpiration through the cell wall. Additionally, lignin is deposited on specific tissue walls to enhance mechanical support and water impermeability (Ninkuu et al., 2023; Bang et al., 2022; Li et al., 2022).

Osmotins and osmotin-like proteins (OLPs) are widely found in plants and named for their ability to adapt to low osmotic environments. This family of proteins belongs to the pathogenesis-related protein 5 (PR5) and is responsive to biotic and abiotic stressors (Chowdhury et al., 2017; Hakim et al., 2018; Bashir et al., 2020). Initial studies have shown that osmotins could be induced by salinity and drought conditions and adapt well to low water potential (Hakim et al., 2018; Bashir et al., 2020). Further research has demonstrated that osmotins and OLPs mediate plant tolerance to abiotic stress. Overexpression of *SnOLP* in soybean significantly improved the physiological state and plant yield under water shortage (Weber et al., 2014). *SindOLP* overexpression promoted the recovery of sesame plants after prolonged exposure to drought and salt stress (Chowdhury et al., 2017). Osmotin-expressing seedlings of apoplastically secreted tobacco osmotin in cotton slowed down the wilting rate following the termination of dry conditions (Parkhi et al., 2009). The above evidence indicates that osmotin family proteins play a protective role against drought conditions.

There are a few reports on osmotins or osmotin-related proteins in rice in terms of stress tolerance. The rice osmotin gene *OsOSM1* conferred immunity against rice sheath blight (Xue et al., 2016). Overexpression of the osmotin protein TlOsm from *Tripogon loliiformis* in rice enhances cold, drought, and salinity tolerance (Le et al., 2018). Overexpression of osmotin promoter binding protein 1 (OPBP1) from tobacco in rice enhances salt stress and defense against belly rot (*Rhizoctonia solani*) and rice blast (*Magnaporthe oryzae*) (Chen and Guo, 2008). The above reports indicate that osmotins or osmotin-related proteins can affect rice stress response. However, relatively little research has been conducted on osmotins or OLPs from rice. There are more than 40 osmotins or OLPs in the rice genome (https://www.genome.jp), designing further studies to characterize their roles in drought tolerance would provide an insight into attempts for drought-tolerant rice transformation.

This study reported a new osmotin-like protein, OsOLP1, identified from rice and demonstrated its involvement in mediating drought tolerance through CRISPR/Cas9 knockout and overexpression techniques. The results showed that overexpression of *OsOLP1* gene enhanced drought tolerance through ABA accumulation, which caused the closure of leaf stomata and the proline enrichment, to alleviate water loss in rice. In addition, OsOLP1 promoted the lignification of vascular tissue and maintained the balance between water and ion distribution. In contrast, CR-*OsOLP1* knockout lines showed more sensitivity to drought. Our data indicated that OsOLP1 is a drought-tolerance protein that can address plant tolerance to external stress.

## Materials and methods

### Plasmid construction, rice transformation, and detection of transgenic plants

The rice gene *OsOLP1* (GenBank accession number: Os01g0839900) was used in the present study. The gene has no intron in its coding region. To generate *OsOLP1* knockout mutant, a gene editing vector for *OsOLP1* was constructed using the CRISPR/Cas9 method (Ma et al., 2015). To improve the targeting efficiency of CRISPR, two sites in the coding region of OsOLP1, T1 (ACCTTGGTCCGGCCGCAT’TGG) and T2 (AGTTGCCGGCCCTCGTGC’GGG) were selected and ligated to *OsU6a* and *OsU6b* promoters by overlapping PCR, and sgRNA was ligated to the end of the target site to construct the “OsU6a-T1-sgRNA-OsU6b-T2- sgRNA” expression cassette, and assembled into the binary pYLCRISPR/Cas9 Pubi-H vectors (digested with *Bsa I*) to generate transgenic rice plants. The target sites and primers (**Supplementary Table 1**) required for vector construction were selected using CRISPR-GE(http://skl.scau.edu.cn/).

To generate *OsOLP1* overexpression line, the full-length coding sequence of *OsOLP1* was amplified from *Oryza sativa* (japonica Nipponbare cultivar) using the All-in-one First-Strand cDNA synthesis SuperMix and TransStart^®^ FastPfu Fly DNA polymerase (TransGene Biotech, Beijing, China) and inserted into the pRHV-cHA (He et al., 2018) vector carrying the *Ubi* promoter and C-terminal HA tag. **Supplementary Table 1** shows the primer pairs required for construction of the vector.

The *Agrobacterium tumefaciens* EHA105 transformation was performed as described previously (Hiei et al., 1994; Clough and Bent, 1998). All constructed vectors were transferred to *A. tumefaciens* EHA105 *via* chemical transformation. 1 μg of recombinant plasmid DNA was added to EHA105 competent cells, placed on ice for 5 min, in liquid nitrogen for 5 min, at 37°C for 5 min, and on ice for 5 min, 700 mL of antibiotic-free LB medium was added and incubated for 2-3 h with shaking, followed by centrifugation to collect the cells, and 100 µL of the suspension was spread on LB plates with 50 μg/mL kanamycin and 25 μg/mL rifampicin. *A. tumefaciens* was co-incubated with callus tissues induced from mature rice seeds, and the desired rice lines were obtained by hygromycin resistance screening and differentiation.

For the identification of CR-*OsOLP1* lines, DNA was extracted from five-week-old leaves, and the coding region of OsOLP1 was amplified using the Cas-*OsOLP1* primer pairs (**Supplementary Table 1**). The PCR products were then sequenced and aligned using DNAMAN. RNA was also extracted from the selected lines (CR-*OsOLP1*#20 and #27), and the expression levels of *OsOLP1* were analyzed by qRT-PCR.

*OsOLP1* overexpression lines (OE-*OsOLP1*) were determined by immunobloting and qRT-PCR. Total protein was extracted from five-week-old leaves in the OE-*OsOLP1* lines using a plant protein extraction kit (Solarbio life science, Beijing, China) according to the manufacturer’s instructions. Rice leaves were extracted with extraction buffer to obtain total protein and then subjected to SDS-PAGE analysis. Samples were incubated with anti-HA antibodies (1:1000) and anti-rabbit (1:5000) and then exposed and photographed with Tanon Chemiluminescence Imaging System. Wild-type seedlings were used as the negative controls. Staining of total proteins with Ponceau S was used as the control. The leaves of the selected lines (OE-*OsOLP1*#28 and #38) were sampled for qRT-PCR analysis. Primer pairs used for the PCR are listed in **Supplementary Table 1**.

### Plant growth and stress treatment

Sterilization of experimental rice seeds consisting of *OsOLP1* overexpressing and *OsOLP1* knockout lines was accomplished by immersing them in 75% ethanol for 15 minutes, and residual ethanol was removed by rinsing seven times. Rice seeds were spread out on a Petri dish containing moistened filter paper and germinated in a growth chamber at 28°C under 16 h light and 8 h in darkness. Sprouted seedlings were transplanted and grown under the same conditions for five weeks. Each pot contains equal amounts of breathable vermiculite and nutrient soil. Samples were quick-frozen in liquid nitrogen and stored temporally at -80°C until analysis.

For the treatment of drought tolerance, five-week-old rice seedlings were continuously deprived of water for 5 days to test for their drought tolerance rate. The seedlings were rewatered for 7 days until the plants recovered, and plant survival was determined by the color and appearance of new leaves. The survival rate was determined by calculating the ratio of total survived seedlings to the total number of seedlings subjected to drought stress. Three replicates of each experiment with 20 seedlings per replicate were performed. To calculate the soil water content (SWC), a 1 ml pipette tip was inserted into the culture soil and vertical sampling of the soil was performed at different (0, 1, 2, 3, 4, 5) days of the drought treatment and SWC was calculated using the formula below. To calculate the relative water content (RWC) of leaves, the full-expanded leaves of *OsOLP1* knockout and overexpression line, and WT were sampled at 0 and 5 days, and RWC was analyzed using the formula below.


$$\text{SWC}\%=\frac{\left(\text{Sf-Sd}\right)}{\text{Sd}}\times 100\;\text{ and RWC}\%=\frac{\left(\text{Wf-Wd}\right)}{\text{(Ws-Wd)}}\times 100$$

where Sf represents soil fresh weight, Sd represents soil dry weight, Wf represents the fresh weight of rice leaves, Wd represents the dry weight, and Ws represents the saturated weight of leaves after immersion in sterile water for 6 h.For the treatment of NaCl, irrigated water was discarded from five-week-old rice seedlings cultured normally, and then the seedlings were watered with water containing 150 mM NaCl. The rice tissues were sampled at different time points to determine the level of gene transcription.

### Total RNA isolation, cDNA synthesis

Triplicate samples of *OsOLP1* overexpression lines, *OsOLP1* knockout lines, and WT were obtained. Total RNA was extracted from related plant tissues (roots, stems, leaves, nodes, and leaf sheaths for *OsOLP1* expression pattern analysis; leaves for CR-*OsOLP1* and OE-*OsOLP1* identification, transcript analysis of ABA and proline synthesis genes) using the EasyPure^®^ Plant RNA Kit (TransGen Biotech, Beijing, China). 1 μg of total RNA was reverse-transcribed into first-strand cDNA using All-in-One First-Strand cDNA Synthesis SuperMix (TransGen Biotech, Beijing, China).

### Quantitative real-time PCR analysis

qRT-PCR primers pairs (**Supplementary Table 1**) were designed using the primer premier 5 and the 2×RealStar Fast SYBR green qRT-PCR Mix purchased from GenStar, Beijing, China. 20 microliters (µl) of the qRT–PCR mix was prepared following the manufacturer’s description, and analysis was carried out using the 96-well desktop QuantStudio 5 real-time PCR system. *Osactin1* (*LOC_Os03g50885*) was used as the internal control for normalization. The relative expression analysis was calculated using the 2^–ΔΔCt^ method as previously performed (Livak and Schmittgen, 2001).

### Quantification of ABA levels

ABA concentration analysis was performed following Ding et al. approach (Ding et al., 2011). About 0.2 g of 3-week-old rice leaves were ground with liquid nitrogen to obtain a fine powder. 500 µL of extraction buffer (90% (v/v) methanol containing 200 mg/L diethydithiocarbamic acid sodium salt) was added and mixed them thoroughly. The extracts were transferred to glass tubes and incubated at 4°C overnight, then centrifuged for 10 min at 8000 g. The supernatant was evaporated to dryness in a new centrifuge tube, and the residue was dissolved with 500 μL of methanol tris buffer. Each experiment included 3 replicates. ABA content was determined using an ABA ELISA Kit following the manufacturer’s instructions (Mlbio, Shanghai, China).

### Scanning electron microscopy of stomata morphology

Five-week-old rice leaves (before and 2 h after drought treatment) were fixed in a fixative buffer containing 2.5% glutaraldehyde overnight at 4°C. The fixative solution was discarded, and samples were washed three times with PBS buffer. After removing excess PBS buffer, samples were desiccated in a serially graded ethanol of 30%, 40%, 50%, 70%, 90%, and 100% for 15 min each. The leaf samples were dried in a vacuum desiccator, mounted on a metal stub with two-sided tape, and sprayed with gold. The stomata were examined using Zeiss Gemini 500 field emission scanning microscope (Gemini 500 FESEM). X500 magnification was used to count the number of stomata per square millimeter, and X1500 magnification to count the percentage of stomata opening and closing from six plants for each line.

### Determination of proline concentration

The proline concentration was determined using Lee et al. method (Lee et al., 2013). 0.2 g of five-week-old leaf tissue was placed in a 2 ml tube and pulverized using a tissue lyser after quickly freezing the samples in liquid nitrogen. 3% sulfosalicylic acid was added to the extract. The samples were boiled at 100°C for 10 minutes and centrifuged at 10,000 g for 10 minutes at room temperature. The supernatant was pipetted into a fresh centrifuge tube, and glacial acetic acid (1 ml) and acidic ninhydrin solutions (1 ml) were added after cooling and after boiling for 1 h. The wavelength of the sample was determined at 520 nm using a spectrophotometer. Each experiment included three replicates. Proline content was calculated using the L-proline concentration standard curve.

### Lignin quantification

Plant growth and preparation were the same as above. The thioglycolic acid (TGA) of lignin content determination method was carried out as previously described (Suzuki et al., 2009). Samples pulverization was the same as performed for proline content analysis. The powdered samples were oven-dried at 60°C for 1 h. 1.8 ml of methanol was added and extracted at 60°C for 20 min. The extract was centrifuged at 16100 g for 10 min at room temperature. The supernatant was discarded, and the extraction step was repeated with methanol. 1 ml of 3 N HCl and 0.1 ml of thioglycolic acid were added, and the samples were heated at 80°C for 3 h and centrifuged at 16100 g for 10 min. The supernatant was carefully removed and discarded, and 1 ml of distilled water was added to the pellet and vortexed for 30 s to mix. The samples were centrifuged as above, and the pellet was resuspended in 1 ml of 1 N NaOH and then vertically rocked for 16 h at 80 rpm. After centrifugation at 16100 g for 10 min at room temperature, the supernatant (about 1 ml) was transferred into a new 1.5 ml tube and acidified with 0.2 ml concentrated HCl. After freezing at 4°C for 4 h, the samples were centrifuged at 16,100 g for 10 min at room temperature. The supernatant was removed, and the pellet was dissolved in 1 ml of 1 N NaOH. After a 50-fold dilution with 1 N NaOH, the absorbance of the solution at 280 nm was measured using the spectrophotometer. Each experiment included four replicates. The lignin concentration was computed using the lignin standard curve as a reference.

### Phloroglucinol-HCl staining of lignin

Cross-sections of rice roots were stained as previously described (Pradhan Mitra and Loque, 2014). The cross-sections were obtained from the roots of five-week-old rice seedlings and incubated in 50% alcohol for 2 h. The sections of root tissues were embedded in an embedding medium, then cut into 100 mm sections with a cryostat, and stained with phloroglucinol-HCl solution (one volume of 37 N HCl to two volumes of 3% phloroglucinol in ethanol). The prepared sections were observed and photographed using the OLYMPUS BX61 microscope at 10X magnifications.

### Data analysis

The amino acid sequence, signal peptide, and disulfide bond analysis of the protein were performed by phytozome (https://phytozome-next.jgi.doe.gov/) and Uniprot (https://www.uniprot.org/); amino acid sequence alignment by DNAMAN 9; gene expression profiling was performed by RiceXPro (https://ricexpro.dna.affrc.go.jp/) and Rice Expression Database (http://expression.ic4r.org/index); Analysis of the spider plot is performed by Hiplot (https://hiplot.cn/).

Data sets from the experiments were subjected to analysis of variance and graphs plotted on the GraphPad Prism, version 9.0.0. Data significance analysis was performed using one-way ANOVA and Tukey’s multiple comparison test. Each bar represents the means and standard deviations from each observation determined at a 95% significance level.

## Results

### OsOLP1 is an osmotic-inducible protein

**Figure 1:**
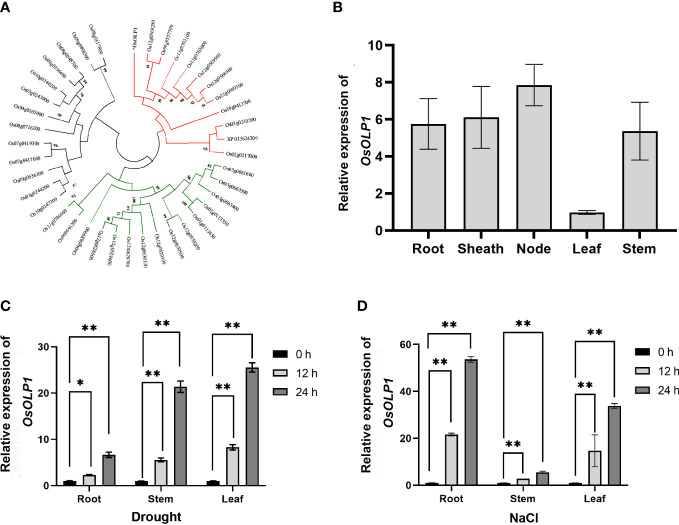
Characteristics and expression patterns of OsOLP1. **(A)** The phylogenetic tree was created using the neighbor-joining method with amino acid sequences of the rice osmotin and osmotin-like proteins. The number of nodes at the phylogenetic tree represents the bootstrap value. **(B)** Quantitative expression of *OsOLP1* in different tissues during vegetative growth in rice. The *Osactin1* was the internal control for quantitative real-time PCR (qRT–PCR) analysis. **(C, D)** show the expression levels of *OsOLP1* in rice after drought and NaCl (150 mM) treatments at 12 h and 24 h, respectively. The *Osactin1* was the internal control for qRT–PCR analysis. Bars denote mean ± SD (n = 3). Asterisks denote the significance level determined by one-way ANOVA followed by Tukey’s multiple comparison tests [*p*≤ 0.05 (*) and *p ≤*0.01 (**)].

To ascertain that OsOLP1 has the characteristics of osmotins and OLPs, we performed cluster analysis of 40 osmotins and OLPs from rice. The result confirmed that OsOLP1 formed a cluster with multiple rice osmotins and OLPs (**Figure 1A**). By Uniprot and phytozome analysis, OsOLP1 was identified as a secreted protein with an N-terminal signal peptide containing 22 amino acids. Without signal peptide, the molecular weight of the mature protein was 24 kDa. The amino acid sequence alignment indicated that the protein has 16 cysteines and is capable of forming eight disulfide bonds (**Supplementary Figure 1**), whose position could be obtained by Uniprot analysis. Furthermore, relative quantification of OsOLP1 revealed transcript abundance in various rice tissues, especially in the leaf nodes (**Figure 1B**). RiceXpro study revealed that *OsOLP1* expression was present throughout the rice life cycle and was highest during the blooming stage. Rice Expression Database exhibited changes in OsOLP1 under various stresses, and environment Ontology indicated that OsOLP1 was associated with desiccation/dehydration stress response. (**Supplementary Figure 2**). Therefore, Drought and NaCl were used to treat rice to explore whether the transcript levels of OsOLP1 respond to osmotic stress. After rice seedlings were exposed to drought and NaCl (150 mM) stress for 12 and 24 hours, the expression level of *OsOLP1* was high in the roots, stems, and leaves (**Figures 1C, D**). These results suggest that OsOLP1 is widely expressed in tissues and responds to different osmotic stresses.

### CRISPR/Cas9-mediated mutagenesis and overexpression lines of *OsOLP1*

**Figure 2:**
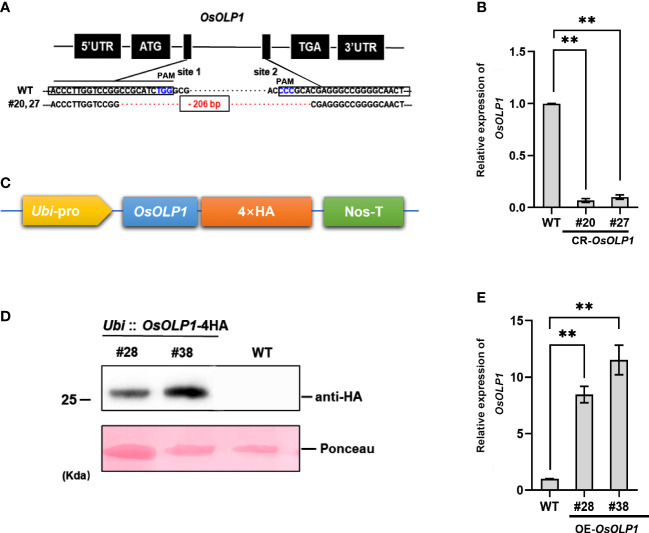
CRISPR/Cas9-mediated mutagenesis and overexpression of *OsOLP1*. **(A)** *OsOLP1* was knocked out by CRISPR/cas9 editing. The black boxes show the targeting sequences, site 1(T1) and site 2(T2), which are driven by promoters and linked to sgRNA for targeting. Both sites are located in the coding region of *OsOLP1*. The PAM area is shown in blue; The 206 base pairs deleted in CR-*OLP1*#20 and #27 are shown in red. **(B)** The relative expression level of *OsOLP1* in CR-*OsOLP1*#20 and #27 lines. The bars denote mean ± SD (n = 3). **(C)** Schematic diagram of the vector pRHV driven by the *Ubi* promoter and containing a 4HA tag at the C-terminus for *OsOLP1* overexpression. **(D)** The expression of *OsOLP1* in OE-*OsOLP1* was analyzed using anti-HA antibodies with the immunoblot method. **(E)** Analysis of *OsOLP1* expression levels in OE-*OsOLP1* lines by qRT-PCR (#28, 38). The *Osactin1* was used as an internal control for qRT-PCR analysis. Bars denote mean ± SD (n = 3). Asterisks denote the significance level determined by one-way ANOVA followed by Tukey’s multiple comparison tests [*p ≤*0.01 (**)].

The genetic regulatory activities of OsOLP1 in drought-stressed rice seedlings were further ascertained by constructing *OsOLP1* knockout plants by the CRISPR/Cas9 system and overexpressing plants. The *OsOLP1* knockout mutant was generated using two targets in the coding sequence (CDS) regions of *OsOLP1* driven by *OsU6a* and *OsU6b* promoters were ligated to sgRNA to generate sgRNA expression cassettes. Then the cassettes were inserted into the pYLCRISPR/Cas9Pubi-H vector for gene editing in wild-type rice, and 48 transgenic lines were obtained. DNA from all the lines was extracted, and the region *OsOLP1* located was amplified and sequenced to determine knockout efficiency. However, only specific target fragments from three lines, #20, #27, and #31, were amplified. Sequencing analysis showed that all three lines were homozygous with a deletion of 206 base pairs between the two sites (**Figure 2A**). The transcript level of *OsOLP1* determined by qRT-PCR was found to be lower in the *OsOLP1* gene editing lines (CR-*OsOLP1*#20 and #27) (**Figure 2B**); therefore, these two lines were selected for further evaluation of drought stress.

Meanwhile, the coding sequence of *OsOLP1* was fused with a 4HA tag driven by the *Ubi* promoter to generate overexpressing rice plants (**Figure 2C**). The expression of *OsOLP1* in overexpression lines OE-*OsOLP1*#28 and #38 was successfully detected by immunoblot analysis using HA antibodies (**Figure 2D**). *OsOLP1* expression levels of OE-*OsOLP1*#28 and #38 were 7-10-fold higher than that of wild-type (**Figure 2E**). The generation of CR-*OsOLP1* and OE-*OsOLP1* helped us to evaluate further the effect of OsOLP1 on drought tolerance in rice.

### OsOLP1 contributes to drought tolerance in rice

**Figure 3:**
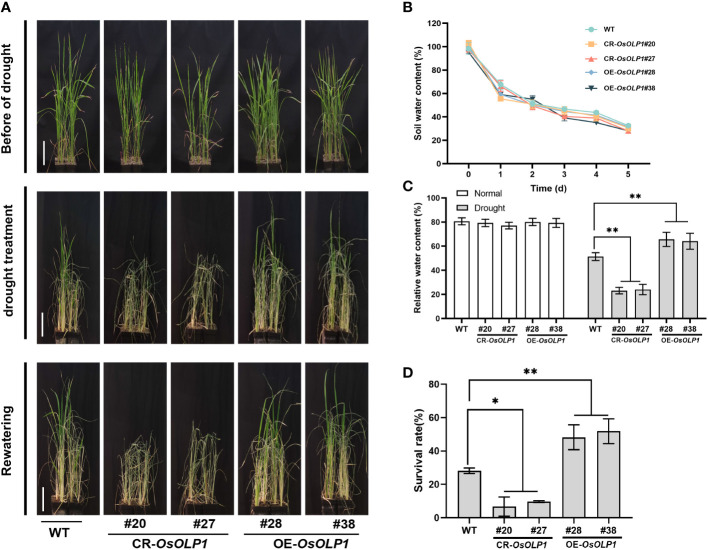
OsOLP1 contributes to drought tolerance in rice seedlings. **(A)** WT, CR-*OsOLP1*, and OE-*OsOLP1* lines phenotypes before drought treatment, after exposure to drought for five days, followed by water recovery. Scale bar, 10 cm. **(B)** Soil water content at different time points under drought stress. Data denotes mean ± SD (n =5). **(C)** Relative water content of leaves of WT, CR-*OsOLP1*, and OE-*OsOLP1* plants before and after drought stress. Data denotes mean ± SD (n =4). **(D)** Survival rates of WT, CR-*OsOLP1*, and OE-*OsOLP1* lines were calculated after rewatering. Data denotes mean ± SD (n = 3). Asterisks denote the significance level determined by one-way ANOVA followed by Tukey’s multiple comparison tests [*p* ≤ 0.05 (*) and *p ≤*0.01 (**)].

Although OsOLP1 also conforms to the structural characteristics of osmotin-like proteins, its role in drought conditions still needs to be verified. Five-week-old seedlings from the CR-*OsOLP1* and OE-*OsOLP1* lines were removed from the irrigation water and subsequently subjected to a 5-day drought without watering. After 5 days, the soil water content was reduced to about 30%, and the plants were in a severe state of water loss (**Figure 3B**). The survival rates of rice were calculated after recovery of 7 days of watering. After five days of drought treatment, the wild type showed survival rates of 28%. Compared to wild type, CR-*OsOLP1* knockout lines showed lower average survival rates of 8.2%, exhibiting more sensitive to water loss, significant drought-induced symptoms, such as leaf curling and wilting, as well as significantly lower recovery rates after rehydration. However, OE-*OsOLP1* lines grew better and maintained an average high survival rate of 53.1% after rehydration (**Figures 3A, D**). In addition, the relative water content of OE-*OsOLP1* (~65%) was higher than that of WT (51.4%), whereas that of CR-*OsOLP1* was lower (~ 25%) (**Figures 3C**). In a nutshell, *OsOLP1* overexpression can avoid rapid water loss and improve rice drought tolerance.

### OsOLP1 influences ABA accumulation

**Figure 4:**
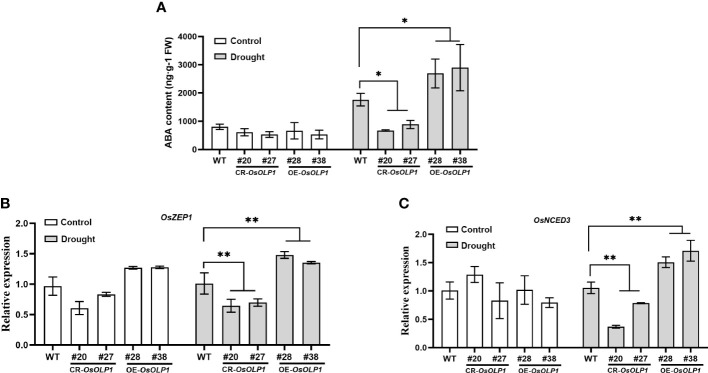
OsOLP1 positively regulated ABA accumulation. **(A)** ABA content in WT, CR-*OsOLP1*, and OE-*OsOLP1* under control and drought-stressed conditions for one day. Data denotes mean ± SD (n = 4). **(B, C)** Transcript abundance of key ABA-encoding genes (*OsZEP1* and *OsNCED3*) in WT, CR-*OsOLP1*, and OE-*OsOLP1* subjected to normal growth conditions and drought treatment. The bars denote mean ± SD (n = 3). Asterisks denote the significance level determined by one-way ANOVA followed by Tukey’s multiple comparison tests [*p* ≤ 0.05 (*) and *p* ≤ 0.01(**)].

ABA can respond to the osmotic stress faced by plants and utilize water rationally. The dehydration signal leads to ABA accumulation and activation of downstream stress-resistance signaling pathways. Therefore, variation in ABA content can be used as the barometer for plant drought tolerance assessment. The ABA content in CR-*OsOLP1*, OE-*OsOLP1*, and WT was analyzed. Under normal condition, no difference in ABA concentration among OE-*OsOLP1*, CR-*OsOLP1* and WT. However, when subjected to a one-day drought, the ABA level in the CR-*OsOLP1* knockout lines decreased by 50%, while that in the OE-*OsOLP1* lines increased by 50% (**Figure 4A**).

However, the question of how OsOLP1 promotes endogenous ABA synthesis was raised. Zeaxanthin epoxidase (ZEP) and 9-*cis*-epoxycarotenoid dioxygenase (NCED) are crucial to ABA synthesis. ZEP1 is the initial enzyme in the ABA synthesis pathway, and NCED3 regulates ABA synthesis under water deficit, and their expression contributes to ABA-mediated drought tolerance. The transcript levels of these two enzymes were quantified by qRT-PCR. The results showed consistency with the ABA content above. The relative transcript abundance of *OsZEP1* and *OsNCED3* in OE-*OsOLP1* lines were significantly up-regulated under drought conditions, while vice versa was observed in CR-*OsOLP1* lines (**Figures 4B, C**). Thus, OsZEP1 and OsNCED3 mediated the OsOLP1-enhanced ABA deposition under drought.

### OsOLP1 modulates stomatal movement under drought

**Figure 5:**
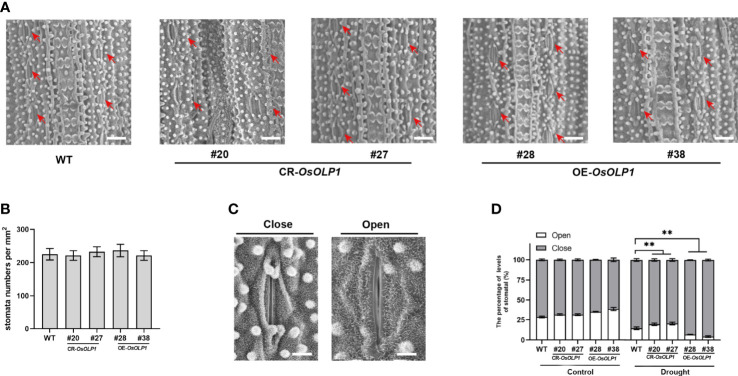
Stomatal distribution and movement in WT, CR-*OsOLP1*, and OE-*OsOLP1* plants. **(A)** Stomatal distribution of WT, CR-*OsOLP1*, and OE-*OsOLP1* plants. The stomatal locations are shown by red arrows. Scale bar, 100 μm. **(B)** Stomatal density per square millimeter was measured in the transgenic plants, CR-*OsOLP1*, and OE-*OsOLP1* lines. Data denote mean ± SD (n = 6). **(C)** Stomatal opening and closing levels of rice were observed by scanning electron microscopy. Scale bar, 10 μm. **(D)** Percentage of stomatal opening and closing of transgenic plants CR-*OsOLP1* and OE-*OsOLP1* lines under normal conditions and after drought exposure. The bars denote the mean ± SD (n = 6). Asterisks denote the significance level determined by one-way ANOVA followed by Tukey’s multiple comparison tests *p* ≤ 0.01(**).

The stomata are enclosed by specialized pairs of guard and subsidiary cells for mechanical support. The stomata regulate water utilization in higher plants through transpiration, and the density and closure of stomata are critical factors for plant drought tolerance. Therefore, we observed the closure and density of epidermal stomata in rice leaves using the scanning electron microscopy technique. As shown in **Figures 5A, B**, there was no significant difference in stomatal density per square millimeter between CR-*OsOLP1*, OE-*OsOLP1*, and WT. Then, after 2 hours of drought exposure, we calculated the percentage of stomatal closure per square millimeter using **Figure 5C** as the standard. The percentage of stomatal closure in CR-*OsOLP1*, OE-*OsOLP1*, and WT lines was not significantly different before drought treatment. However, after drought exposure, the percentage of stomatal closure was 85.6% in the wild type and ~80% in knockout CR-*OsOLP1*, indicating that CR-*OsOLP1* had less stomatal closure than the wild type, while overexpression plants OE-*OsOLP1* showed more stomatal closure numbers of ~96% (**Figure 5D**), suggesting that OsOLP1 can reduce water loss in rice by regulating stomatal apparatus to maintain water balance.

### Overexpression of *OsOLP1* promotes proline accumulation

**Figure 6:**
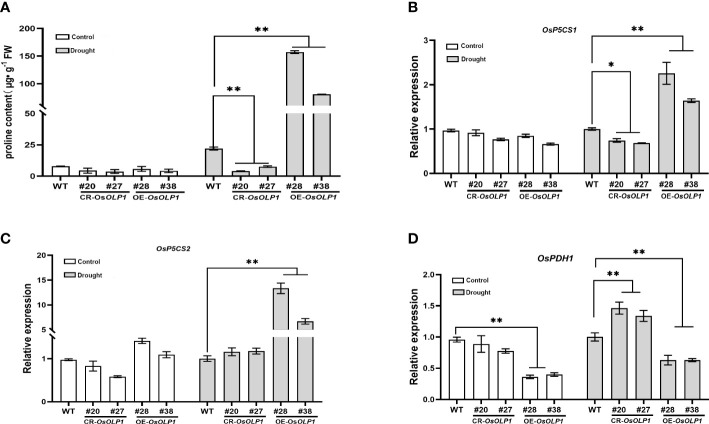
OsOLP1 promotes proline accumulation. **(A)** proline content in transgenic plants CR-*OsOLP1* and OE-*OsOLP1* lines under control conditions and after drought exposure. Bars denote mean ± SD (n = 3). **(B–D)** Expression levels of key proline regulatory genes involved in synthesis (*OsP5CS1* and *OsP5CS*) and degradation (*OsPDH1*) in transgenic plants, CR-*OsOLP1* and OE*-OsOLP1* under normal and drought conditions. The *Osactin1* was used as an internal control for qRT-PCR analysis. Bars denote mean ± SD (n = 3). Asterisks denote the significance level determined by one-way ANOVA followed by Tukey’s multiple comparison tests [*p* ≤ 0.05 (*) and *p* ≤ 0.01(**)].

When plants suffer from unfavorable external conditions, they usually adapt by accumulating protective components. Among these protective components, proline accumulation is characteristic for plant stress defense. It was determined that the proline level was low under normal conditions. However, compared with WT, its levels rapidly increased by 2.7-6.1-fold in the *OsOLP1* overexpression lines and decreased by 1.9-4.4-fold in the *OsOLP1* knockout lines under water deficit conditions (**Figure 6A**). Consistent with this result, the transcript levels of genes encoding delta-1-pyrroline-5-carboxylate synthase (OsP5CS1 and OsP5CS2) involved in the proline synthesis were significantly increased by about 1 to 10 folds in OE-*OsOLP1* lines (**Figures 6B, C**). In addition, the transcription level of the proline-degrading protein proline dehydrogenase (OsPDH1) was significantly increased in CR-*OsOLP1* lines compared to WT (**Figure 6D**). Proline accumulation levels in our report are partly attributable to these regulatory genes’ expression.

### Overexpression of *OsOLP1* contributes to lignin deposition

**Figure 7:**
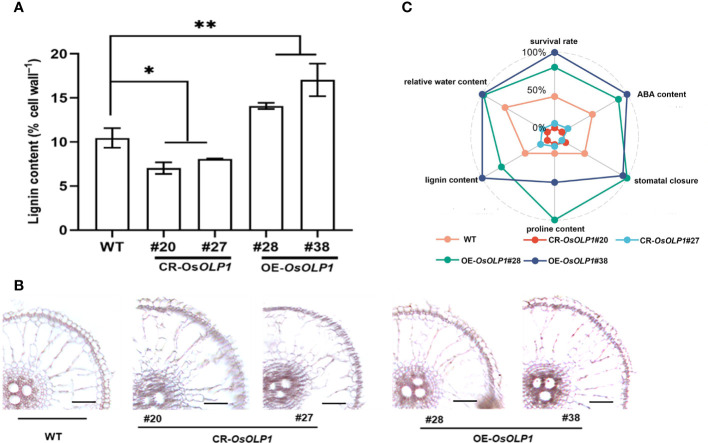
Lignin accumulation in WT and transgenic plants of CR-*OsOLP1* and OE-*OsOLP1*. **(A)** Lignin content was determined in five-week-old seedlings of WT and transgenic plants CR-*OsOLP1* and OE-*OsOLP1*. The bars denote the mean ± SD (n = 4). **(B)** The transverse sections of WT and transgenic plant CR-*OsOLP1* and OE-*OsOLP1* showed roots stained with phloroglucinol-HCl. The vascular tissue of OE-*OsOLP1* lines showed highly lignified. Asterisks denote the significance level determined by one-way ANOVA followed by Tukey’s multiple comparison tests [*p* ≤ 0.05 (*) and *p* ≤ 0.01(**)]. **(C)** A spider plot was used to describe the levels of ABA, proline, lignin, stomatal closure, relative water content, and survival rate in OE-*OsOLP1*, CR-*OsOLP1* and WT under drought condition.

Deposition of lignin leads to the lignification of plant tissues, which often strengthens plants against abiotic stresses. The degree of lignification is proportional to the lignin content. We measured the lignin content of CR-*OsOLP1*, OE-*OsOLP1*, and WT lines using the Thioglycolic acid (TGA) method. The results revealed that *OsOLP1* deletion adversely affects lignin content in rice. However, the lignin concentration of the *OsOLP1*-overexpression lines was significantly 1.3-1.6-fold higher than that of WT (**Figure 7A**). Lignin-specific (Phloroglucinol-HCl) staining of rice roots also visually showed the degree of lignification of CR-*OsOLP1* and OE-*OsOLP1* lines. The sclerenchyma tissues and vasculature in OE-*OsOLP1* lines were highly lignified, whereas WT and CR-*OsOLP1* lines were less lignified (**Figure 7B**). Taken together, OsOLP1 may have mediated the synthesis of lignin.

Finally, a spider plot was used to normalize the effects of OsOLP1 on rice survival rate, relative water content, ABA and proline contents, stomatal closure, and lignin accumulation under drought conditions (**Figure 7C**). The evenly distributed spokes on the wheel showed that CR-*OsOLP1*#20 and *CR-OsOLP1*#27 were closer toward the center (0%), and therefore weakened their tolerance to drought stress. However, OE-*OsOLP1*#28# and OE-*OsOLP1*#38 showed strong drought tolerance as most of their spokes were far from the center on the wheel.

## Discussion

The process of drought tolerance is an integrated response in which plants use various metabolites, hormones, and signaling molecules to activate a series of downstream components through receptor sensing, transduction, and transcriptional regulatory cascades to ensure plant growth and reproduction in arid environments (Singh et al., 2022). Traditional breeding relies on natural genetic variation in related plants, which is rice’s primary strategy for drought-tolerant breeding. At the same time, the development of genomics and precise gene editing tools have made it possible to manipulate plant traits in a targeted manner, which has also greatly improved plant yields under stressful environments (Gupta et al., 2020). Here, *OsOLP1* was investigated as a rice drought tolerance gene that can be used as a complement to the high-quality genetic resources of rice.

Similar to Bowman-Birk trypsin inhibitors, osmotins are cysteine-rich proteins (CRPs), such proteins are evolutionarily conserved to promote plant growth and coordinate stress tolerance (Marshall et al., 2011; Liu et al., 2017; Srivastava et al., 2021). It has been shown that osmotins are involved in plants’ biotic and abiotic stress regulation. Osmotin levels were reported to be maintained at low levels in cells without osmotic treatment (Singh et al., 1985; Anil Kumar et al., 2015). OsOLP1, an osmotin-like protein, is natively expressed in various tissues and abundantly induced in treatments with low water potential (drought and salinity). Kononowicz et al. have reported that the osmotin promoter is spatiotemporally specific in mediating the development of plants after exposure to stress (Kononowicz et al., 1992; Raghothama et al., 1997). We also found that drought and salinity induced different transcriptional profiles of *OsOLP1*, which may be related to the osmotin promoter region and activity (**Figures 1C, D**). Rice, a water-demanding crop, is particularly sensitive to changes in osmotic pressure in the external environment and is often exposed to stresses such as drought in nature. Osmotin-like proteins have also been reported to improve drought tolerance in plants (Weber et al., 2014; Chowdhury et al., 2017). In rice, osmotin-like proteins have not been extensively studied as a genetic resource for drought tolerance. We knocked out and overexpressed *OsOLP1* based on gene editing and genetic transformation techniques and found that overexpression of *OsOLP1* could significantly increase rice survival rate and reduce water loss under drought conditions (**Figures 2**, **3**). Prolonged drought can lead to senescence, abscission of leaves, and even death of the entire plant, which severely hampers crop quality and yield. OsOLP1 also partially delayed or alleviated the development of these symptoms in rice.

The ABA signaling pathway is essential in the drought regulation of plants. The receptor elements of plants sense drought signals causing the accumulation of ABA, and the ABA receptor is phosphorylated and binds to other co-receptors to participate in the regulation of downstream resistance genes by transcription factors (Zhu, 2016; Takahashi et al., 2020). We observed that overexpression of *OsOLP1* under drought conditions increased endogenous ABA content, which is necessary to improve drought tolerance in rice (**Figure 4A**). Transcript abundance of ZEP1 and NCED3, two rate-limiting enzymes of ABA synthesis, was also increased (**Figures 4B, C**), and it has been reported that ZEP and NCED can enhance drought tolerance in plants by regulating ABA synthesis (Iuchi et al., 2001; Park et al., 2008; Zhang et al., 2008; Hwang et al., 2010; Frey et al., 2012; Tong et al., 2017). Similar results have been shown in other studies involved in the overexpression of drought-regulated genes, such as Pyrabactin resistance1-like/Regulatory components of ABA receptors 10 (OsPYL10) (Verma et al., 2019), chloroplastic β-glucosidase isoenzyme (Os3BGlu6) (Wang et al., 2020) and other drought-inducible proteins, such as OsASR1 (Park et al., 2019). These studies proposed that OsOLP1 stimulates the expression of ABA synthesis genes and thus enhances rice’s tolerance to water loss stress. In addition, an osmotin OSM34 from Arabidopsis can interact with an F-box SKP2A in the ABA response pathway, suggesting that osmotin not only mediates ABA synthesis but also is responsible for ABA signaling pathway (Park and Kim, 2021). In contrast, a PR5-like receptor kinase, AtPR5K, which belongs to the same family as osmotin, controls plant drought tolerance by negatively regulating the ABA signaling pathway (Baek et al., 2019), and the reason for this difference is related to the phosphorylation of PR5K, whereas osmotin does not have a phosphokinase domain.

Under drought conditions, plant roots first sense the water loss in the soil. That signal is transported from roots to plant’s above-ground parts *via* CLE25 peptide, and leaves regulate drought resistance dependent on stomatal closure (Christmann and Grill, 2018; Gupta et al., 2020). Stomata are located at the interface between the plant leaves and the atmosphere, and stomatal closure restricts the inflow of carbon dioxide and transpiration to reduce water loss (Matsuda et al., 2016; Durand et al., 2019; Lu et al., 2020). The degree of circulation between stomata and the environment can be described by density and degree of aperture. EPIDERMAL PATTERNING FACTOR 1 (OsEPF1) is responsible for maintaining the density and spacing of stomatal precursor cells, and overexpression of OsEPF1 significantly reduced stomatal density and conductance, further enhancing crop tolerance and yield in drought and high temperature (Caine et al., 2019). We observed that knockout and overexpression of *OsOLP1* had little effect on stomatal density and mainly affected stomatal closure (**Figure 5**), thus avoiding water loss due to transpiration, which was similar to the manner of OsTF1L (Bang et al., 2019). Closure of stomata is a rapid response to ABA synthesis after plants face drought, so we speculate that OsOLP1 positively regulates ABA synthesis to promote stomatal closure and thus further reduce the damage caused by water loss.

Solute accumulation in the cytoplasm maintains cell expansion pressure and promotes water absorption, a strategy that plants use for drought tolerance. Proline is an osmolyte that has been extensively studied under various adverse conditions, and its accumulation is of great significance for plant drought resistance. Furthermore, proline is not only an osmoprotectant but also functions as a metal chelator, antioxidant defense molecule, and signaling molecule, which also contributes to the stress tolerance of higher plants (Ghosh et al., 2022). Increased intracellular proline content has been observed in the overexpression of drought-tolerance genes, such as *ornithine delta-aminotransferase* (*OsOAT*) and *ORANGE* (*OR*) (You et al., 2012; Ali et al., 2022). Osmotin overexpression in transgenic tobacco, carrots, and olives showed high proline accumulation under osmotic stress (Barthakur et al., 2012; Annon et al., 2014; Silvestri et al., 2017). P5CS is a rate-limiting enzyme for the synthesis of proline, and *AtP5CS1* has been identified to be induced and activated by an ABA-dependent regulatory pathway in response to osmotic stress (Savoure et al., 1997; Strizhov et al., 2003; Szabados and Savoure, 2010). However, PDH transcription can be inhibited by dehydration and activated by rehydration, thus preventing proline degradation during abiotic stresses (Kiyosue et al., 1996; Szabados and Savoure, 2010). We suggested that OsOLP1-mediated enhancement of drought tolerance in rice is caused by ABA-regulated proline accumulation, which avoids drought damage through intracellular solute accumulation and reactive oxygen species scavenging.

Controlling the hydraulic conductivity of vascular tissues through lignin accumulation can be seen as another protective mechanism of plants against drought (Zhou et al., 2020). Lignin is a cell wall-strengthening secondary metabolite produced by the phenylpropanoid pathway. Lignin maintains the mechanical support of plants and reduces cell wall water leakage and evaporation (Monties and Fukushima, 2005; Bang et al., 2022). Transgenic rice overexpressing Cinnamoyl-CoA reductase *10* (*OsCCR10*) and *OsTF1L* showed drought tolerance by promoting lignin accumulation (Bang et al., 2019; Bang et al., 2022). Similar results were found in other crops, such as the PdNF-YB21 transcription factor in poplar, IbLEA14 in sweet potato, PoCCoAOMT in tobacco, and VlbZIP30 in grapevine (Park et al., 2011; Tu et al., 2020; Zhou et al., 2020; Zhao et al., 2021). Our study revealed that *OsOLP1* deletion reduced lignin accumulation, whereas overexpression of *OsOLP1* increased lignin content and enhanced the lignification of vascular tissues (**Figure 7**). Therefore, OsOLP1 may promote water balance and plant rigidity by increasing lignin levels and delaying the damage caused by drought.

## Conclusion

**Figure 8:**
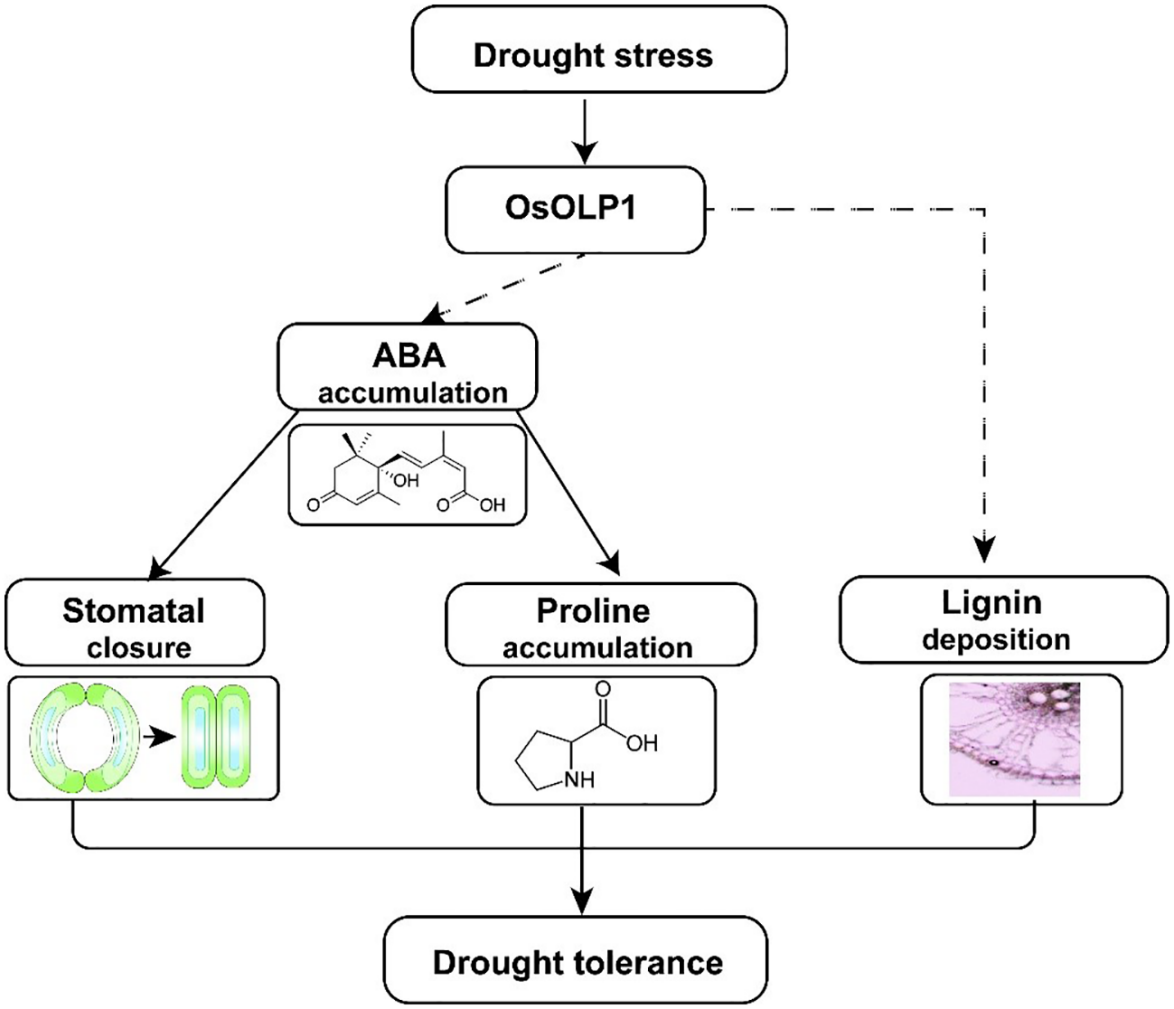
A model illustrating how OsOLP1 mediates drought tolerance in rice. Under drought conditions, up-regulated expression of OsOLP1 activated the ABA synthesis pathway, resulting in the accumulation of ABA, which acts as a signaling molecule to promote the closure of rice epidermal stomata and the synthesis of intracellular proline, thereby reducing water loss and maintaining cell expansion pressure. In addition, OsOLP1 also caused the deposition of lignin, which would make rice better drought tolerance.

In the present study, we identified a new osmotin-like protein, OsOLP1, which has a positive regulatory effect on drought tolerance in rice. OsOLP1 induced the accumulation of endogenous ABA under drought conditions, activated the expression of downstream tolerance genes through the ABA signaling pathway, further reduced water loss by closing stomata, and increased proline content to maintain cellular osmotic pressure. In addition, OsOLP1 directly or indirectly regulates lignin accumulation to enhance plant vascular tissue toughness and ensure water transport (**Figure 8**). However, the regulatory mechanisms of OsOLP1 on ABA synthesis and lignin deposition need to be investigated in depth. The effect of OsOLP1 on rice yield under drought conditions should also be further clarified in the field. The elucidation of the function of OsOLP1 will provide a new perspective for rice stress tolerance breeding.

## Data availability statement

The original contributions presented in the study are included in the article/**Supplementary Material**. Further inquiries can be directed to the corresponding author.

## Author contributions

HZ and JY designed the project, VN, ZF, and TY prepared the materials and data analysis, JY conducted the experiments and drafted the manuscript, and JR, GL, XY, and HZ revised the manuscript. All authors read and approved the final manuscript.
