# Supplementary material for: OsOLP1 contributes to drought tolerance in rice by regulating ABA biosynthesis and lignin accumulation
Source: Front Plant Sci. 2023 May 30;14:1163939. doi: 10.3389/fpls.2023.1163939 (PMC10266352; doi:10.3389/fpls.2023.1163939)
Supplement: Supplementary file 1 [file DataSheet_1.pdf]

Supplementary Material

OsOLP1 contributes to drought tolerance in rice by regulating ABA biosynthesis and lignin accumulation

Jianpei Yan, Vincent Ninkuu, Zhenchao Fu, Tengfeng Yang, Jie Ren, Guangyue Li, Xiufen Yang, Hongmei Zeng*

State Key Laboratory for Biology of Plant Diseases and Insect Pests, Institute of Plant Protection, Chinese Academy of Agricultural Sciences, Beijing, China

*** Correspondence:**
Corresponding Author
zenghongmei@caas.cn

1 Supplementary Figures and Tables

1.1 Supplementary Figures

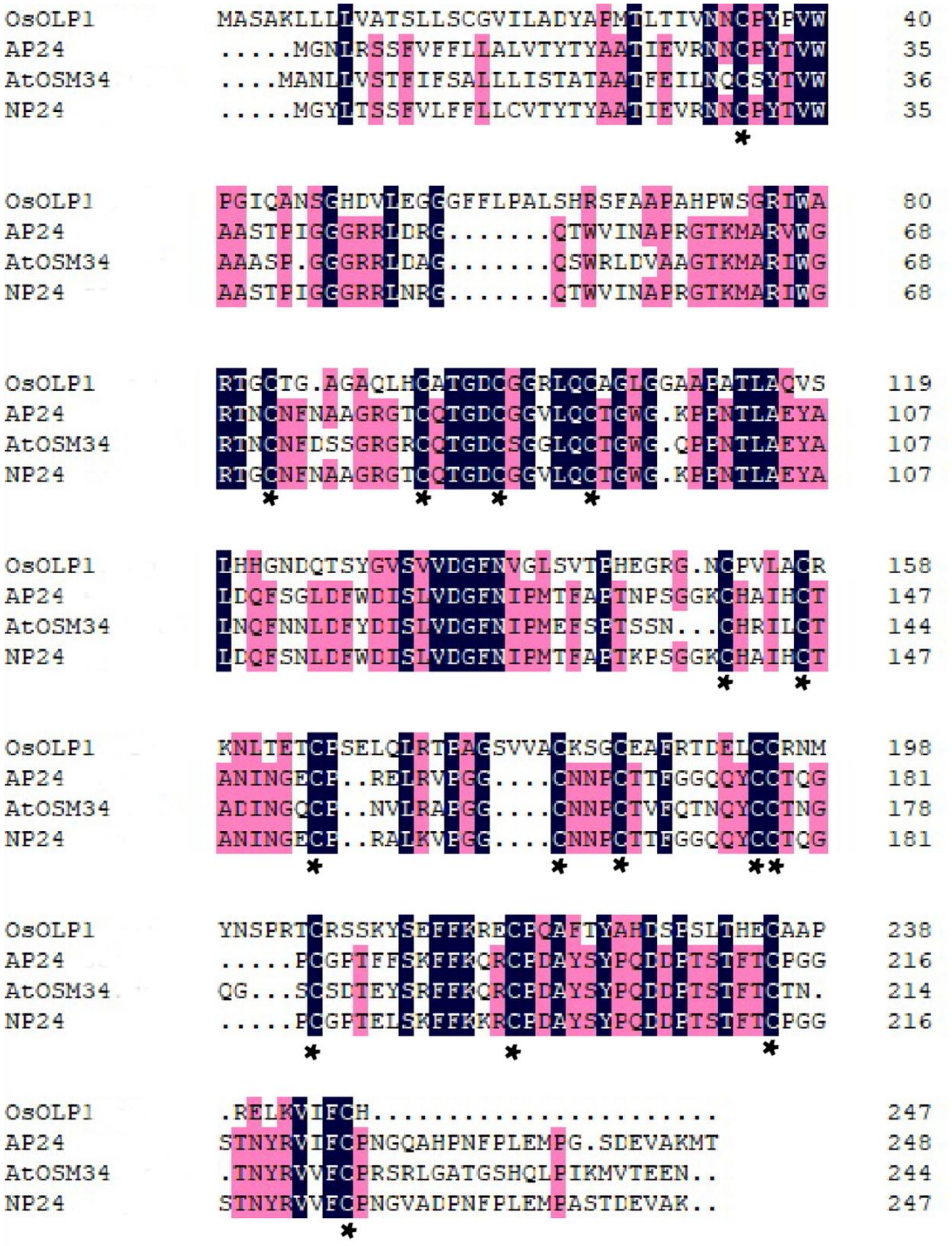

Supplementary Figure 1. The amino acid sequence of OsOLP1 is aligned with the reported osmotin proteins. AP24: an osmotin from *Nicotiana tabacum* (common tobacco). AtOSM34: an osmotin-like protein from *Arabidopsis thaliana*. NP24: an osmotin in *Solanum lycopersicum* (tomato). The asterisk indicates cysteine.

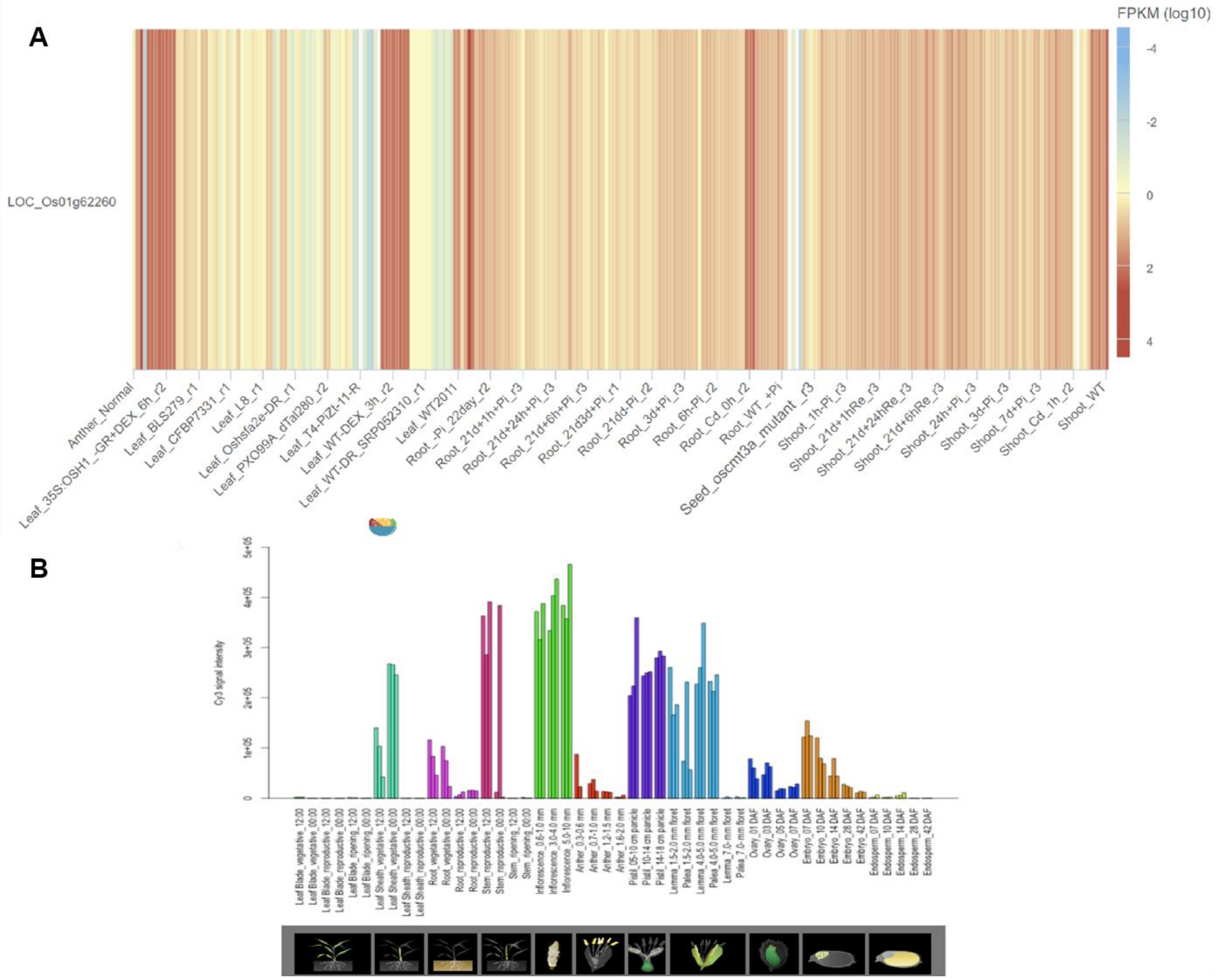

Supplementary Figure 2. Gene expression profiling of OsOLP1 analyzed by RiceXPro **(A)** and Rice Expression Database **(B)**. OsOLP1 is broadly expressed in multiple tissues at different growth stages and environments.

1.2 Supplementary Tables

Supplementary Table 1. Primers used in this study

Primer name	Sequence (5'-3')	Description
OsOLP1-gRT1	ACCCTTGGTCCGGCCGCATCgttttagagctagaaat	CRISPR/Cas9-mediated mutagenesis of *OsOLP1*
OsOLP1-OsU6aT1	GATGCGGCCGGACCAAGGGTCggcagccaagccagca	
OsOLP1-gRT2	AGTTGCCCCGGCCCTCGTGCgttttagagctagaaat	
OsOLP1-OsU6bT2	GCACGAGGGCCGGGGCAACTCaacacaagcggcagc	
U-F	CTCCGTTTTACCTGTGGAATCG	
gR-R	CGGAGGAAAATTCCATCCAC	
OE-OsOLP1-F	tatccagatccagtgggatccATGGCTTCTGCCAAGCTGC	Overexpression of *OsOLP1*
OE-OsOLP1-R	gcggccgcactagtaagcttGTGGCAGAAGATGACCTTGAGC	
Cas-OsOLP1-F	AAACCACGACAACAACAATG	PCR and sequencing analysis of CR-*OsOLP1*
Cas-OsOLP1-R	GCAATAAACGGGTGAACG	
qOsOLP1-F	AACGACCAGACCTCCTACGG	qRT-PCR
qOsOLP1-R	GTCTCGGTCAGGTTCTTGCG	
qOsactin-F	TTATGGTTGGGATGGGACA	

qOsactin-R	AGCACGGCTTGAATAGCG	
qOsPDH1-F	GGTTCCAGGTGAGCAAGT	
qOsPDH1-R	AGACGAGAGCAATCCCCT	
qOsP5CS1-F	TAATGACAGTTTAGCAGGAC	
qOsP5CS1-R	ATACAACCCATCCACATC	
qOsP5CS2-F	CCACCTAGCGATCCACAA	
qOsP5CS2-R	CATTCCACCTCTTCCCAC	
qOsZEP1-F	TCTGGGACCATTGTCGTTTTT	
qOsZEP1-R	GCTCAACATCAAAGGCATTCC	
qOsNCED3-F	CGGAGATGGTGTGGGTGG	
qOsNCED3-R	CGGTCGTCGGACTCGTTG	
